# Running with Scissors: a Systematic Review of Substance Use and the Pre-exposure Prophylaxis Care Continuum Among Sexual Minority Men

**DOI:** 10.1007/s11904-022-00608-y

**Published:** 2022-06-14

**Authors:** Michael Viamonte, Delaram Ghanooni, John M. Reynolds, Christian Grov, Adam W. Carrico

**Affiliations:** 1 Department of Public Health Sciences, University of Miami Miller School of Medicine, 1120 NW 14th St., Office 1010, Miami, FL 33136 USA; 2 Calder Memorial Library, University of Miami, FL Miami, USA; 3 City University of New York Graduate School of Public Health and Health Policy, New York, NY USA

**Keywords:** Adherence, Chemsex, Cocaine, Methamphetamine, Persistence, Pre-exposure prophylaxis

## Abstract

**Purpose of Review:**

Patterns of sexualized drug use, including stimulants (e.g., methamphetamine) and chemsex drugs, are key drivers of HIV incidence among sexual minority men (SMM). Although pre-exposure prophylaxis (PrEP) mitigates HIV risk, there is no consensus regarding the associations of substance use with the PrEP care continuum.

**Recent Findings:**

SMM who use substances are as likely or more likely to use PrEP. Although SMM who use stimulants experience greater difficulties with daily oral PrEP adherence, some evidence shows that SMM who use stimulants or chemsex drugs may achieve better adherence in the context of recent condomless anal sex. Finally, SMM who use substances may experience greater difficulties with PrEP persistence (including retention in PrEP care).

**Summary:**

SMM who use stimulants and other substances would benefit from more comprehensive efforts to support PrEP re-uptake, adherence, and persistence, including delivering behavioral interventions, considering event-based dosing, and providing injectable PrEP.

## Introduction

For over two decades, patterns of sexualized drug use (SDU) have emerged worldwide as prominent drivers of HIV incidence among sexual minority men (SMM) — gay, bisexual, and other men who have sex with men [1–5]. This is evident in early findings from a cohort of SMM in Sydney, Australia, which showed that the use of methamphetamine and erectile dysfunction drugs was associated with an eightfold faster rate of HIV seroconversion from 2001 to 2007 [6]. Similarly, findings from a cohort of SMM in Bangkok, Thailand indicated that methamphetamine use and HIV were intertwining epidemics that disproportionately affected younger men and those who found casual partners on the internet [3]. Another recent study of over 9000 SMM from 7 European countries estimated that nearly 20% reported engaging in SDU and 5% reported using chemsex drugs, including mephedrone, methamphetamine, or gamma hydroxybutyrate (GHB) [2]. In the same study, chemsex drug use was associated with the greatest elevations in multiple indicators of HIV risk [2]. Although some evidence showed the declining use of mephedrone and GHB in the UK [7], SDU and chemsex drug use increased in a cohort of SMM in Amsterdam, Netherlands, from 2008 to 2018 [8]. Other recent findings from this Dutch cohort demonstrated that SMM classified as engaging in chemsex drug use had 15-fold greater odds of a sexually transmitted infection (STI) relative to those reporting no substance use [9]. Taken together, expanded HIV prevention efforts are needed for SMM engaging in SDU and chemsex drug use worldwide.

In the USA, longstanding patterns of SDU are linked to HIV incidence among SMM. Distinct combinations of stimulants (e.g., methamphetamine and powder cocaine), amyl nitrites (i.e., poppers), and erectile dysfunction drugs accounted for nearly two-thirds of HIV infections in a national cohort of SMM from 1998 to 2008 [4]. Currently, a resurgent epidemic of methamphetamine use among SMM in the USA is disproportionately affecting Black and Latino SMM [10–13]. Although methamphetamine use appeared to decline moderately in the USA after significant public health attention in the early to mid-2000s [14, 15], it is again on the rise [16–21]. The enduring consequences for the HIV epidemic are evidenced by recent findings from a cohort of sexual and gender minorities who have sex with men, showing 1 in 3 new HIV infections over 12 months occurring among those reporting methamphetamine use [5]. Although methamphetamine use continues to be strongly associated with engagement in condomless anal sex (CAS) [22, 23], emerging evidence shows that men who use methamphetamine and other stimulants display altered rectal cytokines/chemokines [24, 25]. Bearing in mind that 70% of all HIV infections among SMM occur during receptive CAS [26–28], the confluence of receptive CAS and dysregulated rectal immune function could explain heightened vulnerability to HIV among SMM who use stimulants.

To optimize HIV prevention efforts, there is a clear need to understand the dynamic interplay of structural, social, and psychological determinants of SDU among SMM. High rates of SDU and its social acceptability in urban SMM communities increase exposure risk through social and sexual networks [29–32]. Many SMM reside in concentrated neighborhoods within urban centers where the public health impact of SDU is amplified. This is compounded by sexual minority stress processes (e.g., discrimination) that affect the health of SMM [33–38], which can serve as obstacles to seeking HIV prevention services and substance use disorder treatment [39]. Consistent with the Cognitive Escape Model [40], SDU can function as a means of avoidant coping to manage sexual minority stress and HIV-related stress [41]. Guided by the syndemics theory, multiple psychosocial health comorbidities have also been shown to co-occur with SDU to synergistically heighten HIV risk among SMM [42–47]. For example, there is a dose–response association between a greater burden of syndemic conditions (i.e., stimulant use, polydrug use, heavy alcohol use, depression, and childhood sexual abuse) and faster HIV incidence among SMM [48]. Furthermore, it is well-established that difficulties in managing sexual thoughts, urges, and behaviors (i.e., sexual compulsivity) commonly co-occur with SDU and other syndemic conditions to amplify HIV risk among SMM [42, 49–51]. A recent meta-analysis of studies conducted with SMM revealed a modest association between sexual compulsivity with substance use (*r* = 0.09, SE = 0.02) and a medium association of sexual compulsivity with CAS (*r* = 0.13, SE = 0.02) [52]. Further research is needed to characterize the multi-level determinants of the intertwining epidemics of SDU and HIV in order to guide more comprehensive public health approaches to HIV prevention for SMM.

### HIV Prevention Interventions for SMM Who Use Substances

There is a clear need for integrated pharmacologic and behavioral approaches for the treatment of stimulant use disorders, which are often chronic and relapsing conditions that require ongoing treatment. Placebo-controlled trials conducted to date have focused almost exclusively on methamphetamine use disorder given its association with markedly amplified risk for HIV infection [5]. Although mirtazapine and injectable naltrexone with oral bupropion have shown some promise for the treatment of methamphetamine use disorder [53–55], there is currently no sufficient evidence of the efficacy of pharmacotherapies in treating methamphetamine or cocaine disorders across trials [56, 57]. Most placebo-controlled trials have indicated modest rates of adherence to oral pharmacotherapies, with many integrating the delivery of pharmacotherapies with evidence-based behavioral treatments, such as cognitive-behavioral therapy. At this time, behavioral interventions are considered first-line treatments for SMM who use stimulants such as methamphetamine [58], and these can be delivered across the spectrum of stimulant use disorder symptom severity, including those engaging in binge and episodic patterns of use.

Behavioral interventions have demonstrated efficacy for SMM who use substances, but novel approaches are needed to maximize the benefits of pre-exposure prophylaxis (PrEP) [59]. Contingency management (CM), motivational interviewing (MI), and cognitive-behavioral interventions have demonstrated efficacy in decreasing substance use and CAS among SMM [59–61], but these effects are often modest and short-lived. The persistent nature of HIV risk in this population is supported by findings where SMM who use methamphetamine have fivefold greater odds of receiving a repeat prescription for post-exposure prophylaxis (PEP) and a threefold greater rate of HIV seroconversion [62]. Although supporting entry or re-entry into the PrEP care continuum is an essential first step, it is likely that many SMM who use substances will benefit from additional support for PrEP adherence and persistence. Even with the advent of injectable PrEP [63], efforts to address retention in PrEP care and PrEP persistence will remain essential components of HIV prevention in this high-priority population. It is also unclear whether SMM who use substances will experience difficulties in accessing injectable PrEP when suitable generic oral PrEP formulations are available as daily or event-based dosing [64].

Clinical research examining behavioral interventions to optimize the PrEP care continuum for SMM who use substances has focused mostly on formative and pilot projects to guide the development of interventions [65–68]. However, several randomized controlled trials are underway. These trials are largely focused on adapting and testing evidence-based behavioral approaches, such as CM, MI, and cognitive-behavioral interventions that have previously demonstrated efficacy in reducing substance use and CAS among SMM [59–61].

CM targets extrinsic motivation by providing tangible incentives as positive reinforcement for performing health behaviors [69], and it has also been successfully utilized to promote HIV-related health behavior change in people who use substances [70–75]. CM for stimulant abstinence has demonstrated some benefits for supporting PEP course completion by SMM [76], and ongoing trials are examining the benefits of CM for facilitating (re-)entry into the PrEP care continuum [77], as well as improving PrEP adherence of SMM who inject methamphetamine [78]. Although CM can achieve moderate, short-term reductions in stimulant use [69], there are enduring concerns about the durability of behavior change following the termination of tangible incentives [79]. In our recently completed trial, we observed that delivering a five-session positive affect intervention during CM for stimulant abstinence achieved durable and clinically meaningful reductions in viral load among SMM living with HIV who use methamphetamine [80]. An ongoing trial is examining the efficacy of this positive affect intervention for boosting and extending the benefits of CM for PrEP adherence by SMM who use stimulants [81].

MI is an evidence-based counseling intervention targeting intrinsic motivation for health behavior change that generally yields small but durable improvements in outcomes [79]. Consistent with MI, one recent pilot randomized controlled trial in the STI clinic setting demonstrated the preliminary efficacy of a two-session motivational enhancement and problem-focused intervention compared to treatment as usual for PrEP-naive SMM [82]. Although most men enrolled in this trial reported substance use in the past 12 months, there is a clear need for ongoing trials by our team and others to test MI interventions targeting PrEP use in the context of ongoing SDU and CAS [77, 83, 84].

Cognitive-behavioral interventions are evidence-based approaches to improving adherence in people living with HIV [85]. One pilot randomized controlled trial provided support for the feasibility and acceptability of a six-session, cognitive-behavioral Life-Steps intervention for PrEP adherence [86]. An ongoing trial is testing the efficacy of a stepped care intervention with two-way text messaging in improving PrEP adherence in SMM with one or more syndemic conditions (including stimulant use) [87]. Among participants randomized to the intervention condition, those who do not display improved PrEP adherence in response to text messages will receive the Life-Steps intervention.

### Where are Interventions to Optimize the PrEP Care Continuum Needed in Substance-Using SMM?

Despite the ongoing efforts to develop, test, and implement behavioral interventions to maximize the clinical and public health benefits of PrEP for SMM who use substances, there are fundamental gaps in our understanding of whether and how distinct typologies of SDU and other substance use are linked to difficulties in navigating the PrEP care continuum. SDU broadly encompasses various classes of substances, such as stimulants (e.g., mephedrone, methamphetamine, powder cocaine), poppers, GHB, and erectile dysfunction drugs that are generally used for sexual enhancement motives [41]. SMM also display substantial heterogeneity in their patterns of SDU, ranging from recreational, binge, or episodic use to symptoms consistent with substance use disorders. In this evidence-based review, we had two primary objectives. First, we sought to enumerate studies examining the associations of substance use with the PrEP care continuum: awareness/willingness, use, adherence, and persistence (including retention in care). Second, we evaluated the state of the evidence for the associations of substance use with PrEP use, adherence, and persistence (including retention in care) among SMM. We hypothesized that stimulant use would be more reliably associated with lower PrEP adherence and persistence across studies.

## Methods

We performed a systematic review of the associations of stimulant use with the PrEP care continuum. The search strategy was developed by an academic health science librarian (J.R.) in consultation with the rest of the research team and was reviewed by a medical librarian (see the “Acknowledgments” section) using the Peer Review for Electronic Search Strategies (PRESS) tool [88]. The search strategy was written for Ovid Medline and translated using each database’s syntax, controlled vocabulary, and search fields. MeSH terms, EMTREE, CINAHL, and other subject terms, as well as text words, were used for the concepts of preexposure prophylaxis, PrEP medications, stimulants, substance abuse, drug-related behaviors, and their synonyms. We searched Ovid Medline (including Epub-Ahead-of-Print, In-Process & Other Non-Indexed Citations, and Daily, 1946–present), Embase (Elsevier, Embase.com, 1947–present), Cochrane CENTRAL (Cochrane Library, Wiley, earliest to present), CINAHL with Full Text (Ebsco, 1937–present), Scopus (Elsevier, 1823–present), and the Web of Science platform (Clarivate: Science Citation Index Expanded, Social Sciences Citation Index, Arts & Humanities Citation Index, Conference Proceedings Citation Index-Science, Conference Proceedings Citation Index-Social Science & Humanities, Emerging Sources Citation Index, KCI-Korean Journal Database, Russian Science Citation Index, SciELO Citation Index, BIOSIS, and Zoological Abstracts). No language, date, or other limits were applied at the search phase. The primary search strategy was adapted for other databases in part with the use of the Institute for Evidence Based Healthcare’s Polyglot Search translator [89]. All databases were searched on October 6, 2020 and updated using the same strategies on January 27, 2022. All database records were uploaded to Covidence web-based software for deduplication, screening, and full-text evaluation. We contacted the authors of published abstracts for additional data. One member of our research team (J.R.) checked the Retraction Watch database through EndNote citation management software [90] for retractions of the included studies.

## Results

**Fig. 1:**
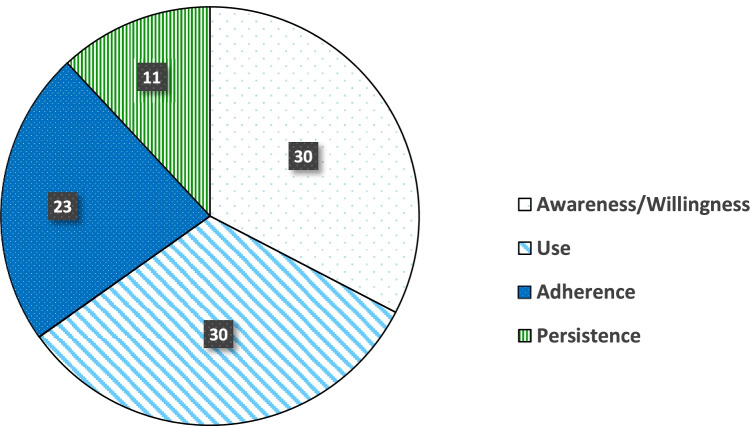
Outcomes examined in studies of the associations of substance use with the PrEP care continuum among sexual minority men

As of January 27, 2022, we identified a total of 4027 articles through our database search after deduplication. Of these, 2397 articles were excluded because (1) the studies did not examine outcomes relevant to the PrEP care continuum, (2) most participants of the studies were not SMM, and (3) the studies did not examine associations of substance use with any indicators of the PrEP care continuum. This resulted in 552 full-text articles that were reviewed by three members of our research team (M.V., D.G., and A.W.C.). Of these full-text articles, 78 were included in our review. As shown in Fig. 1, these studies examined the associations of substance use with 94 outcomes relevant to the PrEP care continuum: awareness/willingness (30), PrEP use (30), PrEP adherence (23), and PrEP persistence (including retention in care, 11).

Thirty studies examined associations of substance use with PrEP use. As shown in Table 1, 18 of these studies (60%) found that SMM who used substances were more likely to use PrEP. Most studies were cross-sectional (*n* = 18), 14 were conducted exclusively in the USA, and none included biomarker confirmation of PrEP use. The findings generally indicated that SMM who used stimulants, such as methamphetamine, and those who engaged in chemsex drug use were more likely to use PrEP. There was also some mixed evidence regarding whether men who used poppers were more likely to use PrEP.

**Table 1:** Studies examining the associations of substance use with PrEP use among sexual minority men (*N* = 30)

Study	Design	Sample	Countries	PrEP use
Blair et al. [98]	Cross-sectional	2020 SMM	Mexico	Chemsex: ↑
Eaton et al. [99]	Cross-sectional	4184 Black SMM	USA	Stimulants: ↑ Poppers: ↑ Erectile dysfunction medications: ↑ Opioids: ↑ Cannabis: ↑ Alcohol use severity: ↑
Feldman et al. [100]	Cross-sectional	102 SMM who use meth	USA (New York City)	Substance use severity: (X)
Flores Anato et al. [101]	Clinic-based cohort	2923 SMM	Canada (Montreal)	Chemsex: (X)
Hambrick et al. [102]	Cross-sectional	444 SMM	France (Paris)	Poppers: (X)
Hammoud et al. [103]	Longitudinal	1257 SMM	Australia	Meth: ↑
Hammoud et al. [22]	Cross-sectional	1367 SMM	Australia	Meth: ↑
Hanum et al. [104]	Longitudinal	1162 SMM	England	Chemsex: ↑
Hardy et al. [105]	Clinic-based cohort	22,255 SMM	Australia (Melbourne)	Substance use during CAS: ↑ Alcohol use during CAS: ↑
Hibbert et al. [106]	Cross-sectional	1648 SMM	England	Sexualized drug use: ↑ Chemsex: ↑
Holloway et al. [107]	Cross-sectional	761 young SMM	USA (California)	Poppers: ↑ Cannabis: (X) Alcohol: (X)
Hoornenborg et al. [92]	Demonstration	374 SMM 2 TGW	The Netherlands (Amsterdam)	Substance use: (X) Substance use severity: (X) Sexualized drug use: (X) Chemsex: (X) Alcohol use severity: (X)
Hulstein et al. [108]	Cross-sectional	5119 SMM 80 TGW	The Netherlands (Amsterdam)	Chemsex: ↑ Meth: ↑ Mephedrone: ↑ GHB: ↑
Khaw et al. [109]	Cross-sectional	416 SMM	Australia	Chemsex: ↑
Kota et al. [110]	Cross-sectional	778 SMM	USA	Substance use severity: (X) Alcohol use severity: (X)
Maksut et al. [111]	Cross-sectional	3429 Black SMM	USA	Lifetime IDU: ↑ Meth: (X) Cocaine: (X) Opioids: (X)
Mansergh et al. [112]	Cross-sectional	841 SMM	USA	Substance use: (X) Alcohol use: ↓
Maxwell et al. [113]	Cross-sectional	165 SMM engaged in chemsex	England	Meth: ↑ Mephedrone: (X) GHB: (X) Mephedrone: (X) Cocaine: (X) Ketamine: (X)
Morgan et al. [114]	Longitudinal	818 SMM 45 TGW 22 gender minority	USA (Chicago)	Substance use: (X) Cannabis: (X) Unhealthy alcohol use: (X)
Onwubiko et al. [115]	Cross-sectional	266 SMM	USA (Atlanta)	Substance use: ↓
Okafor et al. [116]	Cross-sectional	185 SMM	USA (Los Angeles)	Poppers: ↑ Erectile dysfunction drugs: (X) Cannabis: (X) Alcohol use: (X)
Okafor et al. [117]	Demonstration	226 Black SMM	USA	Stimulants: (X) Poppers: (X) Cannabis: (X) Alcohol: (X)
Plotzker et al. [118]	Longitudinal	297 SMM and TGW	Thailand	ATS: (X) Substance use: (X) IDU: (X)
Ramautarsing et al. [119]	Clinic-based cohort	3863 SMM 528 TGW	Thailand	ATS: ↑ IDU: ↑
Serota et al. [120]	Longitudinal	298 Black SMM	USA (Atlanta)	Stimulants: (X) Cannabis: (X) Unhealthy alcohol use: (X)
Schecke et al. [121]	Cross-sectional	1050 SMM	Germany	Meth: ↑
Shover et al. [122]	Clinic-based cohort	18,594 SMM 389 TGW 68 TGM 176 gender minority	USA (Los Angeles)	Stimulants: (X) Poppers: ↑ Erectile dysfunction drugs: ↑ GHB: ↑ Polysubstance use: ↑ Unhealthy alcohol use: ↓
Wheeler et al. [123]	Demonstration	178 Black SMM	USA	Substance use: (X) Polysubstance use: (X) Cannabis: (X) Alcohol use: (X)
Whitfield et al. [124]	Cross-sectional	96,243 SMM	USA and Puerto Rico	Substance use: ↑ Alcohol use: ↑
Wong et al. [125]	Cross-sectional	3043 SMM	Hong Kong	Chemsex: ↑

*ATS* amphetamine-type stimulants, *CAS* condomless anal sex, *GHB* gamma hydroxybutyrate, *meth* methamphetamine, *IDU* injection drug use, *PrEP* pre-exposure prophylaxis, *SMM* sexual minority men, *TGM* transgender men, *TGW* transgender women, (*X*) non-significant results, *↑* significantly greater, *↓* significantly lower

Thirty-three studies examined the associations of substance use with PrEP adherence or persistence (see Table 2). Approximately half (*n* = 16) were conducted exclusively in the USA. Demonstration projects or clinic-based cohorts were the most common designs (*n* = 18). Although 12 studies included biomarkers of PrEP adherence (most commonly tenofovir-diphosphate), only 4 included biomarkers of alcohol or substance use. There were 18 studies that examined associations of stimulants, chemsex drug use, or club drug use with PrEP adherence. More than two-thirds of these studies (*n* = 13) found that stimulants, chemsex drugs, or club drug use were associated with lower PrEP adherence. In contrast, three studies documented associations of stimulant use or chemsex drug use with *better* PrEP adherence, particularly in the context of recent CAS. There was no evidence that cannabis use was associated with lower PrEP adherence. Stimulant use, cannabis use, and substance use were associated with decreased PrEP persistence (including retention in PrEP care) in 5 of the 11 studies. One study showed that SMM with an alcohol use disorder had lower odds of PrEP persistence.

**Table 2:** Studies examining the associations of substance use with PrEP adherence and persistence among sexual minority men (*N* = 33)

Study	Design	Sample	Countries	Adherence	Persistence/retention in care	Biomarkers
Coyer et al. [126]	Longitudinal	365 SMM 2 TGW	The Netherlands (Amsterdam)	–	Chemsex: (X) IDU: (X) Substance use severity: (X) Alcohol use severity: (X)	–
De Franca et al. [127]	Cross-sectional	167 SMM	Brazil	Polysubstance use: ↓	–	–
Flores Anato et al. [101]	Clinic-based cohort	1935 SMM	Canada (Montreal)	–	Chemsex: (X)	–
Goodman-Meza et al. [128]	Demonstration	283 SMM	USA (Los Angeles)	Stimulants: ↓ 4 weeks Stimulants x CAS-MP: ↑ 48 weeks	–	PrEP
Grinsztejn et al. [129]	Demonstration	425 SMM 25 TGW	Brazil	Stimulants: ↑ 48 weeks	–	PrEP
Grov et al. [96]	Cross-sectional	104 SMM	USA (New York City)	Club drugs: ↓ Alcohol: (X) Cannabis: (X)	–	–
Hoenigl et al. [130]	RCT	394 SMM 3 TGW	USA (S. California)	Substance use severity: (X) Meth: (X) Cocaine: (X) Stimulants: (X) Non-stimulants: (X) Heroin: (X) Poppers: (X) Alcohol use severity: (X) Alcohol: (X)	–	PrEP
Hoenigl et al. [131]	RCT	122 SMM and TGW	USA (S. California)	Substance use severity: ↓ post-trial Stimulants: (X) Poppers: (X) Alcohol use severity: (X) Alcohol and cannabis: (X)	–	PrEP
Hoagland et al. [132]	Demonstration	425 SMM 25 TGW	Brazil	Substance use: (X) 4 weeks Binge drinking: (X) 4 weeks	–	PrEP
Hojilla et al. [133]	Clinic-based cohort	268 SMM	USA (San Francisco)	–	Stimulants: (X) Binge drinking: (X)	–
Hojilla et al. [134]	Demonstration	330 SMM and TGW	Peru, Ecuador, Brazil, South Africa, Thailand, and USA	Stimulants: ↓ 4 weeks Binge drinking: (X)	–	Substances PrEP
Hojilla et al. [135]	Demonstration	358 SMM 42 TGW	Peru, Ecuador, Brazil, South Africa, Thailand, and USA	Moderate/Heavy cocaine: ↓ 12 weeks Light cocaine: ↓ 12 weeks	Moderate/Heavy cocaine use: ↓ 72 weeks Light cocaine use: (X)	Substances PrEP
Holtz et al. [136]	RCT	176 SMM 2 TGW	Thailand (Bangkok)	Stimulants: ↓ 30 weeks	–	–
Jin et al. [137]	Demonstration	9,586 participants (91% SMM)	Australia	Meth: ↓ Meth x STI: ↓	–	–
Kota et al. [110]	Cross-sectional	778 SMM	USA	–	Substance use severity: (X) Alcohol use severity: (X)	–
Krakower et al. [138]	Clinic-based cohort	663 participants (96% SMM)	USA (Boston)	–	Substance use disorder: ↓ Alcohol use disorder: ↓	–
Monteiro et al. [139]	Demonstration	320 SMM 18 TGW	Brazil	Stimulants: ↓ Binge drinking: (X)	–	–
Mounzer et al. [140]	RCT	5,313 SMM 74 TGW	Austria, Canada, Denmark, France, Germany, Ireland, Italy, UK, and USA	Meth: (X) Cocaine: (X) Mephedrone: (X) Ecstasy: ↓ GHB: (X) Ketamine: ↓ Poppers: ↓ ED medication: (X)	–	PrEP
Mugo et al. [141]	Cross-sectional	62 SMM	Kenya	Any substance use: (X) Any alcohol use: (X)	–	–
Myers et al. [142]	Demonstration	238 young SMM	USA (N. California)	Substance use severity: (X) Alcohol use severity: (X)	–	PrEP
O’Halloran et al. [143]	RCT	388 SMM	UK	Chemsex: (X)	–	–
Okafor et al. [117]	Demonstration	178 Black SMM	USA	Stimulants during CAS: ↓ 52 weeks Stimulants: (X) Poppers: (X) Alcohol: (X) Cannabis: (X)	–	PrEP
Roux et al. [144]	Demonstration	331 SMM	France and Canada	Chemsex: ↑ during CAS	–	–
Scott et al. [145]	Clinic-based cohort	230 SMM 44 TGW 74 other PrEP	USA (San Francisco)	–	Substance use: ↓	–
Serota et al. [120]	Longitudinal	131 young Black SMM	USA (Atlanta)	–	Stimulants: (X) Cannabis: ↓ Unhealthy alcohol use: (X)	Substances
Shuper et al. [146]	Cross-sectional	141 SMM	Canada (Toronto)	Cocaine use severity: ↓ Unhealthy alcohol use: ↓	–	–
Spinelli et al. [147]	Clinic-based cohort	240 SMM 45 TGW 73 other PrEP	USA (San Francisco)	–	Substance use: ↓ (Excluding cannabis) –	–
Tao et al. [148]	Clinic-based cohort	654 participants (~ 80% SMM)	USA (Providence)	–	Substance use: (X)	–
Vuylsteke et al. [149]	Longitudinal	193 SMM	Belgium (Antwerp)	Chemsex: ↓		–
Wheeler et al. [123]	Demonstration	161 Black SMM	USA	Polysubstance use: (X) Other substance use: (X) Cannabis: (X)		PrEP
Wray et al. [97]	Longitudinal	40 SMM	USA (Providence)	Stimulants: ↓ Stimulants: ↓ in context of CAS Alcohol use severity: (X) Moderate drinking: ↑ in context of CAS Cannabis: (X) Cannabis: ↑ in context of CAS	–	Alcohol PrEP
Wu et al. [150]	Longitudinal	374 SMM	Taiwan	Chemsex: ↓	–	–
Zucker et al. [151]	Clinic-based cohort	696 participants 615 SMM	USA (New York City)	–	Substance use: (X) Alcohol: (X) Cannabis: (X)	–

*CAS-MP* condomless anal sex with multiple partners, *ED* erectile dysfunction, *Meth* methamphetamine, *IDU* injection drug use, *PrEP* pre-exposure prophylaxis, *RCT* randomized controlled trial, *SMM* sexual minority men, *STI* sexually transmitted infection, *TGM* transgender men, *TGW* transgender women, (*X*) non-significant results, *↑* significantly greater, *↓* significantly lower

## Conclusions

In this systematic review, we identified 78 published research articles examining the associations of substance use with indicators of the PrEP care continuum among SMM. Based on this review, we draw three main conclusions. First, SMM who use substances are *as likely* and, in some instances, *more likely* to take PrEP, indicating that this high-priority population does not experience substantial barriers to *accessing* PrEP care. Second, SMM who use stimulants, chemsex drugs, or club drugs can experience greater difficulties with daily oral PrEP *adherence*; however, some evidence shows that SMM who use stimulants or chemsex drugs may achieve *better* PrEP adherence in the context of recent CAS. Third, SMM who use substances may be at greater risk of dropping out of PrEP care or discontinuing PrEP. These findings collectively underscore that SMM who use stimulants and other substances may be “running with scissors” along the PrEP care continuum. Although there seems to be no substantial barriers to initiating PrEP, subsequently, an increased risk of difficulties with adherence and persistence could compromise the benefits of PrEP and increase HIV risk. Taken together, SMM who use stimulants and other substances would benefit from more comprehensive efforts to support PrEP re-uptake, adherence, and persistence, such as implementing evidence-based behavioral interventions, considering the potential benefits of event-based PrEP, and providing access to injectable PrEP.

In contrast to studies conducted predominantly with heterosexual people who inject drugs [91], SMM who use substances do not appear to experience substantial barriers to initiating PrEP. Prevention campaigns targeting SMM have long emphasized the link between the use of methamphetamine or other substances with amplified HIV risk [14, 15]. Among SMM who use substances, PrEP could represent an important harm reduction tool to mitigate the risk of HIV in the context of ongoing SDU and CAS. However, greater recognition of an amplified HIV risk may not be sufficient to mitigate the deleterious associations of substance use with daily oral PrEP adherence and lower PrEP persistence that are observed across studies. Expanded efforts are needed to develop, test, and implement scalable behavioral interventions to support rapid PrEP re-uptake by SMM who use substances.

Our findings have important implications for comprehensive approaches to the improvement of PrEP adherence in SMM who use substances. SMM who use stimulants and club drugs could benefit from behavioral interventions to support their adherence to daily oral PrEP. At the same time, providers should consider the potential benefits of event-based PrEP dosing for SMM who use stimulants and chemsex drugs. Although there is no evidence that SMM who use chemsex drugs display a strong preference for event-based dosing [92], those engaging in episodic or binge patterns of use may be suitable candidates for event-based dosing to improve adherence surrounding CAS episodes. However, SMM who are highly sexually active will likely need daily oral PrEP or injectable PrEP. Further research is needed to guide the implementation of event-based PrEP and injectable PrEP for SMM who use substances.

There is emerging evidence across studies that substance use is linked to difficulties with continued engagement in PrEP care or to lower PrEP persistence among SMM. This underscores the need for behavioral interventions targeting these key behaviors that will remain essential for optimizing HIV prevention efforts among SMM who use substances in the era of injectable PrEP. This is consistent with the longer-term need to support HIV prevention efforts for SMM who use substances. Behavioral interventions for SMM who use substances should focus on re-engagement in PrEP care for those who discontinue PrEP, as well as support for long-term retention in PrEP care and PrEP persistence for those currently taking PrEP.

Transformative HIV prevention approaches for SMM who use substances will require a broader, multi-level framework that delineates intersecting structural, social, psychological, and biological determinants of HIV risk in this high-priority population. It is reductionistic to assume that the observational studies included in this evidence-based review provide evidence for causal, dose–response relationships between substance use and difficulties in navigating the PrEP care continuum. Among SMM, substance use often co-occurs with structural and social determinants of health, such as poverty, housing instability, criminal justice involvement, and intersectional stigma that have important implications for HIV prevention [93]. This is further compounded by multiple, co-occurring syndemic conditions, such as childhood sexual abuse, depression, and sexual compulsivity, which synergistically fuel substance use and HIV risk among SMM [42–47, 49–51]. These intertwining structural, social, and psychological determinants could modify the associations of substance use with difficulties in navigating the PrEP care continuum and amplify rectal immune dysregulation that would increase biological vulnerability to HIV [24, 25]. There is a clear need for prospective, community-based cohorts to examine multi-level factors that moderate or mediate the associations of substance use with difficulties in navigating the PrEP care continuum and heightened HIV risk. This multi-level approach is urgently needed in response to the resurgent methamphetamine epidemic that is fueling one-third of new HIV infections among SMM [5]. Guiding the implementation of more comprehensive public health approaches to maximize the benefits of PrEP for SMM who use methamphetamine is essential to catalyze the success of the “Prevent Strategy” of Ending the HIV Epidemic Initiative in the USA [94].

It is noteworthy that many of the studies included in this review focused on SMM residing in large urban centers, and there is clear evidence that patterns of SDU can vary substantially by region. Future studies should attempt to characterize geographic hotspots for the intersection of SDU and HIV risk among SMM across the globe to guide the targeted deployment of limited public health resources to optimize the benefits of PrEP in this high-priority population. In particular, research is needed to examine the intersection of SDU and HIV risk among SMM residing outside of major urban centers around the globe to inform the development of scalable, telehealth approaches targeting the intersection of SDU and HIV risk. In the USA, SMM who use methamphetamine remain a high-priority population for the Ending the HIV Epidemic Initiative. Developing broader understanding of multi-level determinants of difficulties in navigating the PrEP care continuum and amplified HIV risk is urgently needed to guide transformative HIV prevention efforts in SMM who use methamphetamine.

The findings from this systematic review should be interpreted in the context of some notable limitations. First, due to the substantial heterogeneity in the measurement of distinct typologies of substance use, we were unable to conduct a meta-analysis. Greater consensus is needed in the measurement of specific classes and patterns of substance use across studies. Future studies should attempt to examine distinct patterns of problematic substance use, such as those consistent with a substance use disorder, to identify the relevant threshold(s) where specific classes of substance use have negative consequences for the PrEP care continuum. Although many studies included biomarkers of PrEP adherence, few had biomarkers of recent substance use. Including biomarkers of PrEP and substance use would enhance the scientific rigor of future studies by mitigating misclassification based on self-report. Furthermore, although tenofovir-diphosphate is a validated biomarker of daily oral PrEP adherence, novel approaches (e.g., digital pills) could assist in objectively measuring event-based PrEP adherence [95]. There were also relatively few studies that employed event-level measurements of substance use, PrEP adherence, and CAS [96, 97]. Further research is needed to integrate ecological momentary assessment to better characterize the event-level associations of substance use, PrEP adherence, and CAS. This is particularly important to demonstrate the clinical relevance of PrEP non-adherence that occurs in the context of ongoing CAS (i.e., HIV acquisition risk). Finally, we recognize that other methods of PrEP delivery, including injectable, may soon enter the market on a large scale. Given the novelty of injectable PrEP when this review was conducted, demonstration projects are clearly needed to determine whether and how difficulties in PrEP persistence among SMM who use substances can be addressed in the delivery of injectable PrEP.

This evidence-based review demonstrated that SMM who use substances access PrEP clinical services but could benefit from tailored interventions to support PrEP re-uptake, as well as PrEP adherence, persistence, and retention in care. Multiple randomized controlled trials are testing the efficacy of adapted behavioral interventions to optimize success of SMM who use substances along the PrEP care continuum, and further research is needed to guide the implementation of behavioral interventions that demonstrate efficacy. Providers should also consider the merits of event-based dosing in improving PrEP adherence in SMM who use stimulants or chemsex drugs, as well as provide SMM who use substances with assistance in accessing injectable PrEP.
